# Moult cycle specific differential gene expression profiling of the crab *Portunus pelagicus*

**DOI:** 10.1186/1471-2164-12-147

**Published:** 2011-03-12

**Authors:** Anna V Kuballa, Timothy A Holton, Brian Paterson, Abigail Elizur

**Affiliations:** 1 Faculty of Science, Health and Education, University of the Sunshine Coast, Sippy Downs, Queensland 4556, Australia; 2 Queensland Alliance for Agriculture and Food Innovation, The University of Queensland, St Lucia, Queensland 4072, Australia; 3 Department of Employment, Economic Development and Innovation, Bribie Island, Queensland 4507, Australia

## Abstract

**Background:**

Crustacean moulting is a complex process involving many regulatory pathways. A holistic approach to examine differential gene expression profiles of transcripts relevant to the moulting process, across all moult cycle stages, was used in this study. Custom cDNA microarrays were constructed for *Portunus pelagicus*. The printed arrays contained 5000 transcripts derived from both the whole organism, and from individual organs such as the brain, eyestalk, mandibular organ and Y-organ from all moult cycle stages.

**Results:**

A total of 556 clones were sequenced from the cDNA libraries used to construct the arrays. These cDNAs represented 175 singletons and 62 contigs, resulting in 237 unique putative genes. The gene sequences were classified into the following biological functions: cuticular proteins associated with arthropod exoskeletons, farnesoic acid *O*-methyltransferase (FaMeT), proteins belonging to the hemocyanin gene family, lectins, proteins relevant to lipid metabolism, mitochondrial proteins, muscle related proteins, phenoloxidase activators and ribosomal proteins. Moult cycle-related differential expression patterns were observed for many transcripts. Of particular interest were those relating to the formation and hardening of the exoskeleton, and genes associated with cell respiration and energy metabolism.

**Conclusions:**

The expression data presented here provide a chronological depiction of the molecular events associated with the biological changes that occur during the crustacean moult cycle. Tracing the temporal expression patterns of a large variety of transcripts involved in the moult cycle of *P. pelagicus *can provide a greater understanding of gene function, interaction, and regulation of both known and new genes with respect to the moulting process.

## Background

Moulting is a cyclic process that occurs in all arthropods, from insects to crustaceans, and is essential for growth, reproduction and metamorphosis. The crustacean moult cycle encompasses the period between two successive moults and has been subdivided into 4 major stages; intermoult, pre-moult, ecdysis, and post-moult [1]. The intermoult period is the longest stage of the moult cycle, during which muscle regeneration and the accumulation of energy reserves such as glycogen and lipids occurs [2]. Pre-moult sees the atrophy of somatic muscle, the resorption of the old exoskeleton, and the formation of a new exoskeleton in preparation for the onset of ecdysis [2]. Ecdysis, or the moult itself, involves the shedding of the exoskeleton through a rapid uptake of water from the environment, causing the exoskeleton to rupture [2,3]. Further water uptake occurs during post-moult facilitating the expansion of the new, still soft, exoskeleton; this expansion is essential for the growth of the animal. Exoskeletal hardening, via sclerotization and mineralisation, then takes place [4-6].

Moulting is regulated by an elaborate interplay of hormones, including those which promote, and those which negatively regulate moulting. Among the hormones involved in the induction of moulting are two families of nonpeptidergic hormones: the steroids (ecdysteroids), and the sesquiterpenoids (represented mainly by insect juvenile hormone (JH) and crustacean methyl farnesoate (MF)) [7,8]. Ecdysteroids initiate and coordinate each moult, and are synthesised and secreted by the Y-organs [2]. MF is synthesised by the mandibular organs, and has been implicated in the regulation of crustacean morphogenesis [9,10], metamorphosis [11], reproduction [12-15] and moulting [16]. MF has been shown to directly stimulate the secretion of ecdysteroids in *Cancer magister *Y-organs [16,17]. Additionally, the duration of premoult was significantly reduced in the prawn *Penaeus setiferus *that had been implanted with mandibular organs from *C. magister *[18].

The negative regulatory centre in crustaceans is the sinus gland/X-organ complex, a neurohaemal organ located in the eyestalk [19-22]. The sinus gland/X-organ complex is responsible for the synthesis and storage of a number of inhibitory neuroendocrine hormones, including the moult-inhibiting hormone (MIH), mandibular-organ inhibiting hormone (MOIH) and crustacean hyperglycaemic hormone (CHH) [22]. The MIH of crustaceans continually inhibits ecdysteroid secretion by the Y-organs whereby synthesis of ecdysteroids and subsequent moulting occur only after MIH secretion ceases [21]. CHH, however, plays a multifunctional role as it is central to carbohydrate metabolism, is involved in moult regulation, reproduction, and osmoregulatory function [23-25]. It has been shown to inhibit ecdysteroid synthesis within the Y-organs of *Carcinus maenas *(although not as effectively as MIH) [26]. Furthermore, a synergistic action of suppression of ecdysteroid synthesis in the Y-organ has also been observed to occur when MIH and CHH are incubated together [27]. CHH receptors have been found on Y-organ cells [28], suggesting a physiologically relevant role for CHH in the regulation of ecdysteroid synthesis. CHH has also been shown to influence the iso-osmotic uptake of water during ecdysis, which facilitates body expansion enabling somatic growth [3,25]. Regulation of MF synthesis is negatively controlled by MOIH [29,30], and is thought to occur, in part, through the inhibition of the enzyme farnesoic acid *O*-methyltransferase (FaMeT) that catalyses the final step in the MF biosynthetic pathway [31]. Eyestalk ablation has traditionally been used to induce moulting. This results in a reduction of circulating MIH and therefore promotes the production of ecdysteroids. However, while eyestalk ablation can be effective at inducing moulting, it also leads to lethal ecdysis in some species [32].

Moulting is a complex process that is affected by a range of external and internal factors including temperature, photoperiod, nutritional state and eyestalk integrity. In order to explore the molecular events associated with the moulting process, microarray technology has been implemented to investigate differential gene expression in *Portunus pelagicus *at various stages of the moult cycle. Microarray technology offers the potential to examine the expression patterns of many genes simultaneously, thus gaining a more comprehensive understanding of gene function, interaction, and regulation. This has enabled both the assessment of expression profiles of known genes, and the discovery of new genes that play a role in the moult cycle of crustaceans. *P. pelagicus *(commonly known as the blue swimmer crab) was used as a model species to study moulting as its life cycle has been closed at the Bribie Island Research Centre (BIRC), eliminating the need for wild caught animals.

## Results

### Overview of *P. pelagicus *EST sequence distribution

**Figure 1:**
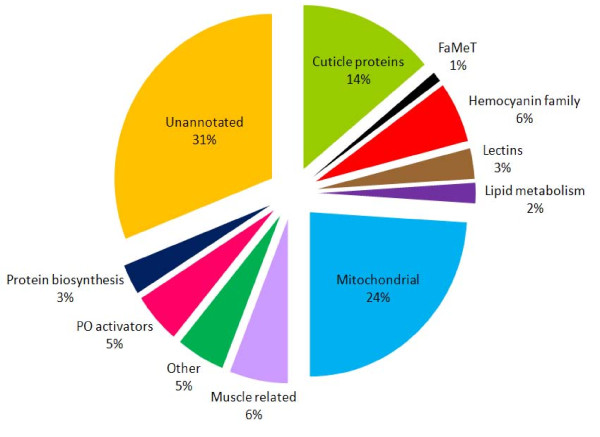
**Distribution of sequenced cDNA transcripts isolated from the entire microarray study, grouped according to biological function**.

A total of 556 clones were sequenced from the cDNA libraries used to construct the *P. pelagicus *cDNA arrays. Prior to array printing, 160 of these were sequenced in order to determine the quality of each cDNA library. Factors such as sequence length and redundancy were considered in the assessment. A 30% redundancy of 16 S rRNA was determined in the initial sequencing stage. The proportion of 16 S rRNA was greatly reduced (from 30% to 3%) by screening the libraries prior to printing the arrays, demonstrating the successful removal of 16 S rRNA from the original cDNA libraries. The majority of the sequencing however, was carried out subsequent to microarray analysis to identify genes that demonstrated differential expression profiles across the moult cycle. 396 clones were randomly selected from a list that displayed differential expression (P ≤ 0.05) patterns between moult stages. This approach enabled the identification of genes likely to be involved in, and important for, crustacean moulting. The 556 cDNAs were assembled in Sequencher based on sequence similarity; this resulted in 175 singletons and 62 contigs, representing 237 unique putative genes. Sequence annotation was via BLASTn, BLASTx and Pfam domain analysis. The expressed gene sequences were grouped according to the following biological functions: cuticular proteins associated with arthropod exoskeletons, FaMeT, proteins belonging to the hemocyanin gene family (cryptocyanin, hemocyanin and metallothionein), lectins (C-type lectin receptor, mannose-binding protein, glycoproteins, mucin and a proline rich protein), proteins relevant to lipid metabolism (fatty acid binding protein and diazepam binding inhibitor), mitochondrial proteins (ATP synthase, cytochrome oxidases and NADH dehydrogenase), muscle related proteins (actin, myosin, and thymosin), phenoloxidase (PO) activators (serine proteases such as trypsin and chymotrypsin, antimicrobial and clotting proteins), ribosomal proteins (including translation and elongation factors), and other sequences that did not fall into these groups. Unannotated transcripts, were so termed, because they displayed no significant sequence similarity with sequences deposited in the NCBI database and were therefore not able to be annotated by BLAST analysis. The percentage distribution of the 556 sequenced cDNAs is depicted in Figure 1.

The largest group of transcripts depicted here (31%) represents cDNAs that could not be annotated via the GenBank database. Transcripts encoding mitochondrial proteins such ATP synthase, cytochrome oxidases and NADH dehydrogenase make up 24% of the total cDNAs isolated in this study. Cuticular protein transcripts constitute 14%, while transcripts of the hemocyanin gene family and those related to muscle function and development comprise 6% each, of the total cDNA population. Phenoloxidase (PO) activators such as serine proteases, antimicrobial and clotting protein transcripts contribute to 5% of all sequenced cDNAs. Other transcripts encoding diverse proteins not classified into the other groups include ovary development-related protein, opsin, ferritin, heat shock protein, tubulin, notch protein, arginine and pyruvate dehydrogenase kinase, and transcripts that contained CT, GT or AC repeats, represent 5% of the total population. Lectins, such as the C-type lectin receptor and mannose-binding protein, as well as ribosomal proteins, each contributed to 3% of all sequenced cDNAs. Fatty acid binding protein and diazepam binding inhibitor transcripts, that are associated with lipid metabolism, constitute 2% of the overall transcript population, while FaMeT transcripts represent the smallest group that form 1% of all cDNAs sequenced within the scope of this microarray study.

### Gene expression profiles across the moult cycle of *P. pelagicus*

**Figure 2:**
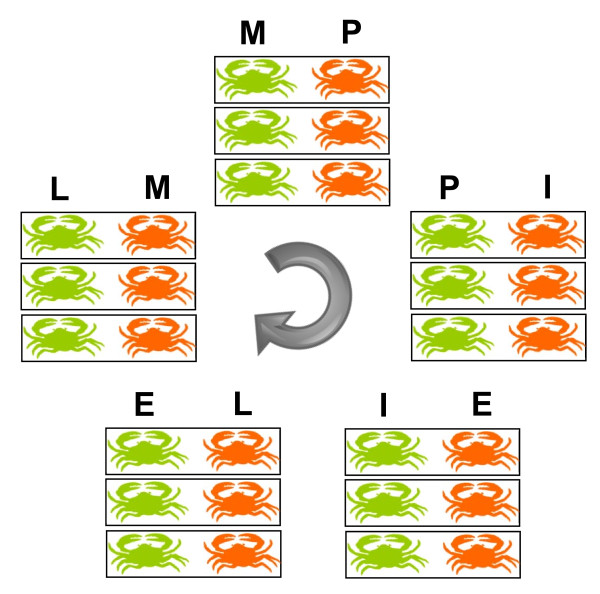
**Experimental design for moult stage hybridisations**. Each rectangle represents an array with 3 individuals pooled from each stage; each array was repeated 3 times with different individuals. The green colour represents the label Cy3 while orange represents Cy5 (M = moult, P = post-moult, I = intermoult, E = early pre-moult, L = late pre-moult).

Moult stage-specific differential gene expression analysis was carried out on *P. pelagicus *crabs from the following moult stages: post-moult, intermoult, early pre-moult, late pre-moult and ecdysis, using custom prepared *P. pelagicus *cDNA microarray slides. A loop design, in which consecutive moult stages were compared via dual channel microarray hybridisations, was employed for the analysis (Figure 2). This format enabled the generation of a time series plot of differentially expressed genes across the moult cycle.

Analysis with GeneSpring using K-means clustering revealed seven main subsets of transcripts that displayed differential expression profiles across the moult cycle of *P. pelagicus*. They were grouped into clusters labelled A-G according to their expression patterns. Not all of the 556 clones selected for sequencing fell into these clusters and many of the transcripts associated with these clusters were not sequenced.

**Figure 3:**
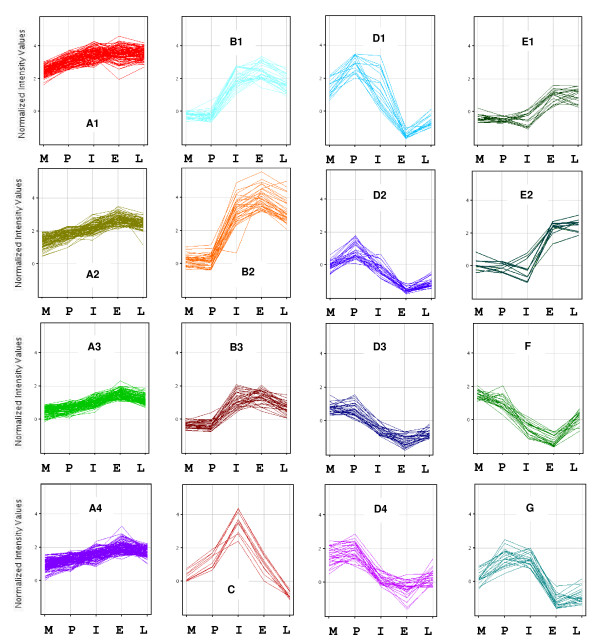
**K-means clustering maps of the collective clusters featuring time series expression profiles which vary according to moult stage**. Fold change in expression is depicted on the vertical axis and moult stages are depicted on the horizontal axis whereby M = moult, P = post-moult, I = intermoult, E = early pre-moult, L = late pre-moult.

The transcripts assigned to Cluster A are relatively down-regulated at the time of moulting, expression subsequently increases at each consecutive stage, peaking in early pre-moult then plateauing or declining slightly in late pre-moult in preparation for ecdysis. In this subset, four clusters (A1-A4) were deemed to have similar expression profiles and hence collectively termed Cluster A; this group is shown in Figure 3. Table 1 describes the composition of Cluster A, referring to each individual cluster respectively (A1-4). The largest proportion (46%) of sequenced transcripts in Cluster A are of mitochondrial origin, 15% are metallothionein, 10% actin, 9% myosin, 8% opsin, 4% ferritin, while ribosomal RNA and elongation factor make up 2.5% each, hemocyanin, chitinase and a CT repeat sequence contribute to 1% of the transcripts sequenced from Cluster A.

**Table 1:** Summary of the genes contained within Cluster A.

Transcript ID - Annotation	Accession #	Transcript #
**Cluster A1**		
*Gecarcinus lateralis *beta-actin (act1) mRNA	L76943	8
*Marsupenaeus japonicus *myosin light chain mRNA	GU584102	7
*Portunus trituberculatus *mitochondrial DNA cytochrome b & cytochrome c oxidase	AB093006	2
*Cancer productus *18 S ribosomal RNA gene, internal transcribed spacer 1,2	EF035125	1
*Portunus pelagicus *opsin mRNA	EF110527	1
*Eriocheir sinensis *ferritin 3 mRNA	GU475115	1
*Portunus pelagicus *hemocyanin (HEM6)	EF110536	1
*Libinia emarginata voucher *Lem elongation factor-2 mRNA	AY305506	1
Unsequenced		92
**Cluster A2**		
*Portunus trituberculatus *mitochondrial DNA ATP synthase, cytochrome c oxidase, NADH dehydrogenase	AB093006	13
*Eriocheir sinensis *ferritin 3 mRNA	GU475115	2
Unsequenced		71
**Cluster A3**		
*Portunus pelagicus *metallothionein (METAL1) mRNA	EF110529	12
*Portunus trituberculatus *mitochondrial DNA ATP synthase, cytochrome b & cytochrome c oxidase, NADH dehydrogenase	AB093006	8
*Portunus pelagicus *opsin mRNA	EF110527	4
*Scylla serrata *chitinase mRNA	EU402970	1
40 S ribosomal protein S12 [*Dermacentor variabilis*]	AAP04352	1
CT repeat		1
Unsequenced		64
**Cluster A4**		
*Portunus trituberculatus *mitochondrial DNA ATP synthase, cytochrome b & cytochrome c oxidase	AB093006	13
*Portunus pelagicus *opsin mRNA	EF110527	1
putative elongation factor 1 beta' [*Diaphorina citri*]	ABG82006	1
Unsequenced		71

Transcript # indicates the number of copies of each gene present within the cluster.

Cluster B consists of transcripts which are down-regulated in the moult and post-moult stages, expression then increases dramatically in intermoult, remains high in early pre-moult and begins to decline in late pre-moult. This group is depicted in Figure 3 where three clusters (B1-B3) were deemed to have similar expression profiles and hence collectively termed Cluster B. Table 2 describes the composition of Cluster B; each subset of Cluster B is referred to individually. Hemocyanin makes up 33% of the sequenced transcript population, cryptocyanin 23%, trypsin 15%, cathepsin 6%, chymotrypsin 5%, carcinin 3%, while fatty acid binding protein, dehydrogenase, ATP synthase, fumarase and arginine kinase each make up 2%. Additionally 6% of the sequenced transcripts remain unable to be annotated.

**Table 2:** Summary of the genes contained within Cluster B.

Transcript ID - Annotation	Accession #	Transcript #
**Cluster B1**		
*Portunus pelagicus *hemocyanin subunit 1, 2, 5, 6, 7 (HEM1, 2, 5, 6, 7) mRNA	EF110531, EF110532, EF110535, EF110536, EF110537	6
*Portunus pelagicus *trypsin (TRY1) & (TRY3) mRNA	EF120993, EF120995	3
*Portunus pelagicus *chymotrypsin-like serine protease (CTRY2) mRNA	EF120997	2
*P.vannamei *cathepsin-L like cysteine protease mRNA	X85127	2
*Portunus pelagicus *carcinin mRNA	EF120999	2
*Portunus pelagicus *cryptocyanin 1 & 2 mRNA	EF102021, EF102022	2
*Pacifastacus leniusculus *intracellular fatty acid binding protein mRNA	DQ459987	1
Unannotated		1
Unsequenced		11
**Cluster B2**		
*Portunus pelagicus *cryptocyanin 1 & 2 mRNA	EF102021, EF102022	12
*Portunus pelagicus *hemocyanin subunit 1, 3, 6 (HEM1, 3, 6) mRNA	EF110531, EF110533, EF110536	9
*Portunus pelagicus *trypsin (TRY1) & (TRY3) mRNA	EF120993, EF120995	4
*Ixodes scapularis *fumarase	XM002411012	1
*Carcinus maenas *arginine kinase (AK) mRNA	AF167313	1
Unsequenced		7
**Cluster B3**		
*Portunus pelagicus *hemocyanin subunit 1, 3, 6, 7 (HEM1, 3, 6, 7) mRNA	EF110531, EF110533, EF110536, EF110537	5
*P.vannamei *cathepsin-L like cysteine protease mRNA	X85127	2
*Portunus pelagicus *trypsin (TRY1) & (TRY3) mRNA	EF120993, EF120995	2
*Portunus pelagicus *chymotrypsin-like serine protease (CTRY2) mRNA	EF120997	1
*Portunus trituberculatus *glyceraldehyde-3-phosphate dehydrogenase	EU919707	1
*Litopenaeus vannamei *mitochondrial ATP synthase subunit alpha precursor	GQ848643	1
Unannotated		3
Unsequenced		20

Transcript # indicates the number of copies of each gene present within the cluster.

Cluster C is a relatively small group of transcripts (ten in total), in which expression is down-regulated in the moult and post-moult stages, increases substantially in intermoult, then drops dramatically in early and late pre-moult (Figure 3). Of the sequenced transcripts from Cluster C, three are cuticle proteins (see Table 3 for sequence IDs).

**Table 3:** Summary of the genes contained within Cluster C.

Transcript ID - Annotation	Accession #	Transcript #
**Cluster C**		
*Portunus pelagicus *cuticle protein CUT7 & CUT8-like mRNA	EF101999, EF102000	3
Unsequenced		7

Transcript # indicates the number of copies of each gene present within the cluster.

The transcript profile that is represented in Cluster D displays expression that peaks in the post-moult stage, decreases dramatically in the intermoult and early pre-moult stages, then begins to increase again in late pre-moult and ecdysis. Figure 3 depicts the four K-means clustering maps that collectively make up Cluster D, while Table 4 describes transcript identity and number. The sequenced transcripts from Cluster D consist of 58% cuticle proteins, gastrolith protein and vermiform cuticle protein each constitute 2%, while 38% were unable to be annotated.

**Table 4:** Summary of the genes contained within Cluster D.

Transcript ID - Annotation	Accession #	Transcript #
**Cluster D1**		
*Portunus pelagicus *cuticle protein BD1, CUT1, CUT2, CUT12, CUT13 mRNA	EF102013, EF101993, EF101994, EF102004, EF102005	11
Unsequenced		7
**Cluster D2**		
*Portunus pelagicus *cuticle protein CUT1, CUT3, CUT4, CUT6, CUT12, CUT13, CB3 mRNA	EF101993, EF101995, EF101996, EF101998, EF102004, EF102005, EF102008	16
Unannotated		4
Unsequenced		12
**Cluster D3**		
*Portunus pelagicus *cuticle protein BD1 & BD2 mRNA	EF102013, EF102014	3
Unannotated		10
Unsequenced		16
**Cluster D4**		
*Portunus pelagicus *cuticle protein CUT13 mRNA	EF102005	1
*Cherax quadricarinatus *gastrolith protein (GAP65) mRNA	EU551670	1
*Portunus pelagicus *vermiform cuticle protein VER3-like mRNA	EF102020	1
Unannotated		6
Unsequenced		18

Transcript # indicates the number of copies of each gene present within the cluster.

The group of transcripts represented in Cluster E display expression profiles which are relatively low during the moult, post-moult and intermoult stages, then increase in the early pre-moult stage and remain high in late pre-moult. This group is depicted in Figure 3 where two clusters (E1 & E2) were deemed to have similar expression profiles and hence collectively termed Cluster E. Table 5 describes the composition of Clusters E1 and E2. In this cluster 31% of sequenced transcripts are fatty acid binding proteins, carcinin and C-type lectin receptor each make up 25%, 13% are vermiform cuticle proteins and 6% of the sequenced transcript population is clotting protein.

**Table 5:** Summary of the genes contained within Cluster E.

Transcript ID - Annotation	Accession #	Transcript #
**Cluster E1**		
*Fenneropenaeus chinensis *fatty acids binding protein mRNA	GQ377108	3
*Portunus pelagicus *carcinin mRNA	EF120999	3
*Portunus pelagicus *putative clotting protein precursor, mRNA	EF120998	1
*Portunus pelagicus *C-type lectin receptor (CTLR) mRNA	EF120992	1
*Portunus pelagicus *vermiform cuticle protein VER2-like mRNA	EF102019	1
Unsequenced		10
**Cluster E2**		
*Portunus pelagicus *C-type lectin receptor (CTLR) mRNA,	EF120992	3
*Fenneropenaeus chinensis *fatty acids binding protein mRNA	GQ377108	2
*Portunus pelagicus *carcinin mRNA	EF120999	1
*Portunus pelagicus *vermiform cuticle protein VER2-like mRNA	EF102019	1
Unsequenced		2

Transcript # indicates the number of copies of each gene present within the cluster.

Cluster F is depicted in Figure 3 and features transcripts whose expression is highest in the moult and post-moult stages, decreases substantially in the intermoult and early pre-moult stages then begins to increase again in late pre-moult in preparation for ecdysis. Table 6 provides a description of the identity and number of the total transcript population for Cluster F. A high proportion of the sequenced cDNAs (38%) are mannose-binding proteins, 23% are cuticle proteins, 8% represent myosin, while 31% remain unannotated.

**Table 6:** Summary of the genes contained within Cluster F.

Transcript ID - Annotation	Accession #	Transcript #
**Cluster F**		
*Portunus pelagicus *mannose-binding protein (MBP1)	EF102990	5
*Portunus pelagicus *cuticle protein BD2 & CBM	EF102014, EF102017	3
*Marsupenaeus japonicus *myosin light chain mRNA	GU584102	1
Unannotated		4
Unsequenced		10

Transcript # indicates the number of copies of each gene present within the cluster.

The expression profiles of the transcripts comprising Cluster G show relatively low expression levels in the moult stage, an increase to peak levels in the post-moult and intermoult stages, then a dramatic decrease in the early pre-moult stage which begins to increase again in late pre-moult. The gene expression pattern for Cluster G is presented in Figure 3 Table 7 describes the identity and number of the transcripts assigned to Cluster G, of which the sequenced transcripts comprise of 50% cuticle proteins and 50% unannotated sequences.

**Table 7:** Summary of the genes contained within Cluster G.

Transcript ID - Annotation	Accession #	Transcript #
**Cluster G**		
*Portunus pelagicus *cuticle protein CUT7 & CB4-like mRNA	EF101999, EF102009	3
Unannotated		3
Unsequenced		14

Transcript # indicates the number of copies of each gene present within the cluster.

**Figure 4:**
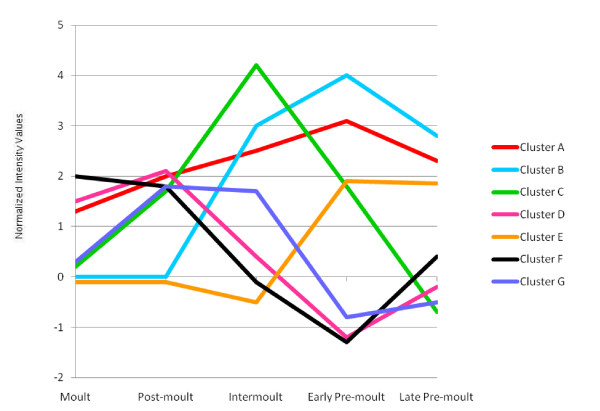
**Overview of the temporal gene expression profiles observed for each cluster across the moult cycle**.

Figure 4 presents a summary of all the expression profiles described above and allows a comparison of cluster profiles. A peak in down-regulation in the early pre-moult cycle can be seen in clusters D, F and G, while a peak in up-regulation in this moult stage is observed in clusters A, B and E.

## Discussion

A holistic approach to gene expression profiling was employed in order to gain a greater understanding of the molecular events associated with the crustacean moulting process. A *P. pelagicus *moult cycle specific cDNA microarray, containing sequences from 5000 cDNA clones derived from whole crabs in addition to individual organs such as the brain, eyestalk, MO and Y-organ from all moult cycle stages, was developed for this study. By comparing the expression patterns of transcripts from each moult cycle stage it was possible to identify genes with potential involvement in various aspects of the moulting process.

Genisphere's 3DNA dendrimer labeling technology, which overcomes some difficulties associated with direct or indirect labelling methods, was used in this study. This technology allows the labelling of cDNA via a target capture sequence oligonucleotide rather than incorporating the fluorescent dye directly during cDNA preparation, thus avoiding inefficient synthesis and hybridization of the cDNA to the array that results from the incorporation of fluorescent dye nucleotide conjugates into the reverse transcript. Additionally, the signal generated from each message will be independent of base composition or length of the transcript (Genisphere 3DNA Array 900MPX protocol).

The sequencing of 556 clones from two libraries (whole crab and crab organ) generated from *P. pelagicus*, revealed that a significant portion of these cDNAs (31%) could not be annotated via the GenBank database (Figure 1). However, these transcripts may nevertheless represent genes with a significant role in the process of moulting in crustaceans, as they were isolated within the scope of the moult cycle-related differential gene expression analysis study.

### Cellular energy requirements across the moult cycle

Transcripts representing mitochondrial proteins, such as ATP synthase, cytochrome oxidase, and NADH dehydrogenase form the second largest group (24%) of cDNAs isolated during this microarray study (Figure 1). Such proteins are required for cellular energy homeostasis as they are a part of the mitochondrial respiratory chain [33]. NADH dehydrogenase and cytochrome c oxidase are two of the three energy-transducing enzymes in the mitochondrial electron transport chain. Mitochondrial (including ATP synthase, cytochrome oxidase and NADH dehydrogenase) ribosomal and elongation factor transcripts, were identified in Cluster A (Figure 3 Table 1) which display an expression profile of relatively low expression during ecdysis then a gradual increase across the rest of the moult cycle generally peaking in early pre-moult then decreasing slightly in late pre-moult. The expression profile of these transcripts appears to reflect an increase in the energy requirements of the animal as the moult cycle progresses. Lower levels of mitochondrial gene expression at ecdysis suggests reduced energy requirements during moulting, with a recovery in metabolic activity appearing in the post-moult stage and returning to normal during interphase, reflected in the increase in mitochondrial gene expression observed in Cluster A. Interestingly, studies into the ecdysteroid responsive genes of *Cherax quadricarinatus *revealed that moult induction through endocrine manipulation resulted in differential expression of genes predominantly belonging to a group known to be involved in metabolic functions such as digestive enzymes, carbohydrate metabolism and mitochondrial respiration [34]. These transcripts however, were down-regulated in pre-moult when compared to control animals in intermoult, possibly indicating that moult induction creates metabolic stress which may impact on metabolic function. Hormonal effects of mitochondrial transcription have also been reported in mammals; thyroid hormones for example stimulate mitochondrial activity as well as gene expression [35]. Moulting in crustaceans is under the hormonal control of ecdysteroids, which in *C. sapidus *peak in pre-moult and return to basal levels in the post-moult stage [36]. The results presented here reflect this pattern of hormonal stimulation of mitochondrial transcription, as the moult cycle progresses in *P. pelagicus*, genes associated with energy production including mitochondrial and ribosomal transcripts, show an increase in expression levels across consecutive stages of the moult cycle.

A similar expression profile occurs for further transcripts of ATP synthase, arginine kinase and fumerase (found in Cluster B, Figure 3). These transcripts show down-regulation in the moult and post-moult stages and then an increase in expression levels in the intermoult and pre-moult stages. Phosphagen kinases (which include arginine kinase and creatine kinase) function in temporal ATP buffering and in intracellular energy transport [37]. Phosphagen kinases are abundant in muscle, where they maintain ATP homeostasis during muscle contraction [38,39], in the gills which function in nitrogen excretion and gas exchange [40] and in cell migration [41]. Interestingly, arginine kinase is the sole phosphagen kinase found in arthropods [42]. The enzymatic activity of arginine kinase in *C. maenas *was found to vary significantly according to tissue type with the highest levels observed in the claw muscle [40]. Given the important role that arginine kinase plays in energy production we postulate that ATP buffering by arginine kinase may occur temporally as well as spatially in order to meet the fluctuating metabolic requirements experienced across the moult cycle. The enzyme fumarase facilitates the production of energy in the form of NADH in the mitochondria. The expression profiles observed here for fumarase, suggest that it could be important in meeting the high energy demands of growth during the moult cycle.

### Cuticular protein expression

Transcripts encoding cuticular proteins represent 14% of the total cDNAs isolated in this microarray study. Several patterns of moult cycle differential expression were observed for cuticular proteins, implying that each group has a specific but varying function depending on which stage of the moult cycle up-regulation is detected. For instance a peak in expression during intermoult was found to occur for cuticle proteins CUT7 and CUT8 (EF101999, EF102000) (Cluster C, Figure 3). This expression pattern was also identified using an independent data analysis method, where up-regulation was observed in intermoult when compared to both post-moult and early pre-moult [43], both of these transcripts code for proteins that contain three cuticle_1 domains. Proteins with the cuticle_1 domain are associated with calcified cuticle in decapod crustaceans [44,45]. The specific up-regulation of these cuticle trancripts in the intermoult phase indicates that formation and/or repair of the exoskeleton may continue throughout the intermoult phase and that these genes operate separately to those involved in the formation of new cuticle during the pre- and post-moult stages. The largest proportion of cuticle protein transcripts (31%) was found to occur in Cluster D. The transcripts identified in this group include *P. pelagicus *cuticle protein genes BD1 (EF102013), BD2 (EF102014), CUT1 (EF101993), CUT2 (EF101994), CUT3 (EF101995), CUT4 (EF101996), CUT6 (EF101998), CUT12 (EF102004), CUT13 (EF102005), CB3 (EF102008), *P. pelagicus *vermiform cuticle protein VER3-like (EF102020), and *C. quadricarinatus *gastrolith protein (GAP65) (EU551670). Here we see an expression profile of up-regulation in the moult and post-moult stages (peaking in post-moult) then a sharp decline during intermoult and early pre-moult, followed by a recovery in the late pre-moult stage. GAP65 was found to be directly involved in the deposition of amorphous calcium carbonate in the gastroliths of *C. quadricarinatus *[46]. Based on the expression profile observed for transcripts VER3 and GAP65 in Cluster D, a role in the calcification of the crustacean cuticle seems likely.

A similar pattern is seen in Cluster F which contains 13% cuticle protein transcripts, composed of *P. pelagicus *cuticle protein BD2 (EF102014) and CBM (EF102017). Transcripts with the abbreviation BD, code for proteins with a PfamB_109992 domain [43] which has yet to be annotated but has been isolated from the calcified cuticle of other crabs [44]. CUT transcripts, when translated, contain the protein domain cuticle_1, also associated with calcified cuticle. CB transcripts, on the other hand, code for proteins with a chitin_bind_4 domain. In addition to its chitin binding function, this chitin binding domain also occurs in proteins which have been demonstrated to function in calcification of the crustacean exoskeleton [47,48]. CMB (chitin binding Peritrophin-A domain) is another transcript group with chitin binding abilities, prevalent in insects and involved in the structural formation of the peritrophic membrane (gut lining in arthropods) [49]; it has also been found in penaeid prawns. Despite the differences in domain type, and hence assumed functional difference, these transcripts follow a synergistic pattern of expression, which displays up-regulation at ecdysis with a peak in post-moult. The high level of expression in post-moult together with functional annotation suggest that these genes are involved in the synthesis and hardening of the post-moult crustacean cuticle. *P. pelagicus *cuticle protein CUT7 (EF101999) and CB4 (EF102009), observed in Cluster G, present with a slightly different profile where expression is highest in post-moult and intermoult, decreases dramatically in early pre-moult then begins to increase again in late pre-moult. The incidence of cuticular protein up-regulation in intermoult, when compared to early pre-moult (Clusters C and G), is perhaps unexpected because the exoskeleton is considered to be fully formed by the intermoult stage. This may indicate a continued synthesis and/or repair of the exoskeleton well into the intermoult period followed by a down-regulation of cuticular protein expression in the pre-moult period, in preparation for the degradation and eventual shedding of the exoskeleton at ecdysis. Stillman also reports a peak in transcript levels of certain cuticle protein genes in the middle of the moult cycle [50].

Additionally, two transcripts that displayed similarity to a low-density lipoprotein receptor class A (LDLa) domain containing chitin binding protein from *Drosophila *(termed VER2 & 3) exhibited two different types of expression profiles, VER2 in Cluster E and VER3 in cluster D. The opposing expression profiles of Clusters D and E (transcripts specific to D display transcripts that show a decrease in expression in early pre-moult when compared to intermoult, whereas Cluster E shows expression that is low in intermoult and high in early pre-moult), together with the specificity of the transcript type identified in each cluster, suggests different physiological roles and modes of action for VER2 and VER3.

### Differential expression of the hemocyanin gene family

Transcripts belonging to the hemocyanin gene family represented 6% of all sequenced cDNAs (Figure 1); these include hemocyanin, cryptocyanin and metallothionein. Moult cycle-related differential expression of hemocyanin and cryptocyanin was evident in Cluster B where high levels of expression are seen in the intermoult and pre-moult stages. Recent studies examining global expression patterns of *C. magister *juveniles also found differential expression patterns occurring across developmental stages for both hemocyanin and cryptocycanin [50]. Hemocyanin is an oxygen transport protein that is found in the hemolymph of crustaceans [51]. In addition to its ability to reversibly bind oxygen, hemocyanin also displays PO activity [52] which is important to the sclerotization or hardening of the newly synthesised cuticle [53]. Hemocyanin has been located in the cuticle of the prawn *Penaeus japonicus *during the intermoult and postmoult stages of the moult cycle. Here the enzymatic activity of cuticular hemocyanin was higher than that of hemocyanin derived from the hemolymph [53]. Additionally, ecdysone (arthropod moulting hormone) has been found to bind to proteins within the crustacean hepatopancreas and cuticle [54]. More recent studies on the tarantula, suggest that this protein may be hemocyanin. The spider hemocyanin was found to bind both ecdysone and 20-OH-ecdysone, albeit with low affinity which is thought to be compensated for by its high concentration [55]. The authors calculated that up to 75% of the ecdysteroids can be transported by hemocyanin. Considering the important role hemocyanin is thought to play in cuticle formation and ecdysone transport, the high levels of hemocyanin gene expression observed in the present study in both the intermoult and pre-moult periods reflect the dual functionality of hemocyanin in preparation for arthropod ecdysis.

Cryptocyanin is structurally related to hemocyanin however it lacks the ability to bind oxygen [56]. Instead cryptocyanin is involved in protein transport and in the formation of the new exoskeleton in crustaceans [56,57]. The similarity in gene expression profiles of crytocyanin and hemocyanin, together with their structural relatedness, suggests a similarity in function with respect to cuticle synthesis, both through direct incorporation and the potential transfer of other cuticular components. Metallothionein on the other hand was up-regulated gradually across the moult cycle (found in Cluster A, Figure 3) where expression was lowest at ecdysis and peaked in early pre-moult. This expression profile was also observed for transcripts associated with mitochondrial energy metabolism such as ATP synthase, cytochrome oxidase and NADH dehydrogenase. Metallothionein is a ubiquitous heavy metal-binding protein, involved in copper homeostasis and detoxification [58]. Studies in *C. sapidus *have demonstrated the presence of metallothionein in pre-moult crabs, suggesting that metallothionein is required for the regulation of biologically available copper ions necessary for the oxygen binding properties of hemocyanin [58]. Crustacean metallothionein has also been implicated in the regulation of energy metabolism by affecting mitochondrial respiration. Investigations on *H. americanus *demonstrated that metallothionein is present in the intermembrane space of hepatopancreatic mitochondria and is able to regulate the oxygen consumption of mitochondria in a zinc-dependant manner [59]. The synchronous expression profile of metallothionein and several genes involved in mitochondrial respiration, observed here in Cluster A, support the hypothesis of a regulatory role for metallothionein in energy production. Metallothionein was also found to exert a protective effect against the highly reactive oxygen species generated by oxygen metabolism in the presence of zinc [59]. Free zinc in quantities equivalent to those tested when bound by metallothionein increased the levels of reactive oxygen species by four fold. Crustaceans have been found to store considerable levels of metals such as calcium, copper and zinc in the hepatopancreas during the pre-moult stage of the moult cycle [60]; moreover induction of metallothionein levels in the hepatopancreas occurs at high zinc concentrations [61]. The accumulation of zinc in the hepatopancreas during pre-moult, together with the role of zinc in inducing oxidative stress, accentuates the requirement for protective measures against free radical formation in this moult cycle stage. The peak of metallothionein expression in pre-moult lends further support to the implied role of metallothionein in metal detoxification and energy metabolism.

### Phenoloxidase activity

PO activators such as the serine proteases trypsin, chymotrypsin, and trypsinogen, in addition to antimicrobial and clotting proteins, made up 5% of the total distribution of sequenced cDNAs (Figure 1). Trypsin and chymotrypsin both displayed moult cycle related differential expression in that they were highly up-regulated in intermoult and pre-moult when compared to ecdysis and post-moult (Cluster B, Figure 3). Trypsin is one of the major digestive proteases secreted by the hepatopancreas [62], chymotrypsin also, is a serine protease recently identified in the digestive systems of crustaceans [63]. Studies on *Penaeus vannamei *revealed that mRNA expression of trypsin is at a maximum during early premoult, then declines sharply in late premoult. The specific activity of trypsin also followed this pattern, suggesting the regulation of trypsin biosynthesis is, at least in part, transcriptional and may be under the influence of ecdysteroid hormones [64]. Further research on the midgut gland of *P. vannamei *showed that mRNA expression of trypsin also differed across the moult cycle [65]. They found a high level of trypsin expression in intermoult, a peak in early pre-moult, followed by a decline in late pre-moult with lowest levels in the post-moult stage; these figures correlate strongly with the results from this study in *P. pelagicus*. Sanchez-Paz and Garcia-Carreno suggested that this expression pattern may be explained through feeding behaviour during the moult cycle, as trypsin is a digestive enzyme and feeding occurs mostly in the intermoult and pre-moult stages. Interestingly, trypsin and chymotrypsin are the only two digestive enzymes that were found to be differentially expressed across the moult cycle in this study, presumably additional digestive enzymes would be up-regulated if these expression profiles were due solely to feeding behaviour. Perhaps a further explanation of trypsin and chymotrypsin activity may be attributed to their roles in the phenoloxidase (PO) cascade. The PO pathway has typically been associated with immunity but is also involved in important structural aspects of the crustacean cuticle such as melanisation and sclerotization [6,66-68]. The PO cascade requires activation which is achieved via several mechanisms including C-type lectins, and the proteases trypsin [69,70] and chymotrypsin [71].

Trypsin and chymotrypsin expression correlates strongly with hemocyanin expression (Cluster B), and may be involved in activation of the PO pathway and the stimulation of hemocyanin into an active phenoloxidase-like enzyme, that is associated with melanin synthesis and sclerotization in the newly developing cuticle in *P. pelagicus*.

### Genes involved in cuticle hardening

Lectins, which include the calcium dependant lectin group (C-type lectin) receptor, mannose-binding protein, mucin and a proline rich protein, represent 3% of the cDNAs isolated in this study (Figure 1). C-type lectin receptor transcripts followed the expression pattern observed in Cluster E, with relatively low levels in moult, post-moult and intermoult then an increase in the pre-moult stages (Figure 3). Conversely, the mannose-binding protein (present in Cluster F) was highly expressed at ecdysis and post-moult.

Glycoproteins, such as the mannose rich variety found in the calcified cuticle of *C. sapidus*, have been found to be associated with the regulation of biomineralisation [72]. Shafer and colleagues describe an alteration in the lectin-binding characteristics of mannose rich glycoproteins at the time of onset of calcification. Glycosylated cuticle proteins are thought to act as pre-moult inhibitors of calcification, deglycosylation of these proteins occurs specifically after ecdysis likely initiating the deposition of calcium [72,73]. In this study both the C-type lectin receptor and the mannose-binding protein display significant moult cycle-related differential expression. The C-type lectin receptor is up-regulated in the pre-moult stages, coinciding with the formation of new cuticle, which must remain uncalcified prior to moulting, while the mannose-binding protein is up-regulated in the moult and post-moult period. The temporally specific, and high levels of, up-regulation of both of these genes have been proposed to be involved in the regulation of calcification in the crustacean cuticle [74].

Lectins (carbohydrate binding proteins) are also involved in immune function through the lectin-complement pathway, in which the mannose-binding lectin recognises infectious agents and triggers PO activation [75,76]. PO activity also plays a role in cuticle sclerotization [66] and melanisation [77]. The up-regulation of mannose-binding protein observed here during periods of cuticle hardening, coupled with its role in the activation of the PO cascade, suggest that it also participates in the sclerotization of the crustacean exoskeleton.

### Muscle formation

Muscle-related cDNAs such as actin, myosin and thymosin, constituted 6% of all the transcripts isolated during the moult cycle-related microarray experiments (Figure 1). Differential expression of muscle-related transcripts was observed across the moult cycle where a gradual up-regulation of actin and myosin transcripts was observed between edcysis and the early pre-moult stage (Cluster A, Figure 3). Actin possesses diverse cellular functions which include the provision of mechanical support in the cytoskeleton, the mechanism for muscle contraction in muscle cells, and the binding of ATP in the cytosol [78]. Myosins are a large family of motor proteins that facilitate actin-based motility, via an interaction with actin and the hydrolysis of ATP [79]. Muscle mass, particularly in the claws of large decapod crustaceans, undergoes cyclic atrophy during pre-moult followed by regeneration during the post-moult and intermoult periods [2,80]. The up-regulation of actin and myosin observed from moult through to early pre-moult is consistent with the observation that muscle deposition and growth occur mainly in the intermoult period.

### Lipid metabolism

Transcripts encoding the lipid metabolism proteins diazepam-binding inhibitor and fatty acid binding protein constituted 2% of all sequenced transcripts (Figure 1). Fatty acid binding protein transcripts were found in Cluster E, where an up-regulation is observed in the pre-moult stages when compared to the rest of the moult cycle. The fluctuation of lipid composition in the hypodermal membrane of the exoskeleton has been demonstrated in several crustacean species. Observations in *C. pagurus *show that the hypodermis increases in lipid content just before secretion of the new exoskeleton begins in pre-moult [81]. Cuticular lipid levels in *C. sapidus *have been shown to increase during pre-moult and peak dramatically post ecdysis before returning to intermoult levels [82]. These cuticular observations reflect the changes detected in the hemocytes of *C. maenas *which become loaded with lipid prior to ecdysis. Hemocytes aggregate beneath the hypodermis and apparently transfer the lipid to the newly forming cuticle [81]. Furthermore, fatty acid binding proteins have been isolated from the hemocytes of the crayfish *Pacifastacus leniusculus *and the prawn *Penaeus monodon *[83]. The moult cycle-related changes to the expression of fatty acid binding protein, demonstrated here, may facilitate the deposition of lipids in the cuticle of crustaceans.

## Conclusions

Tracing the temporal expression patterns of genes involved in the crustacean moult cycle provides a platform for gaining a greater understanding of gene function, interaction, and regulation with respect to the moulting process. The expression data presented here provide a chronological depiction of the molecular events associated with the biological changes occurring during the crustacean moult cycle.

Transcripts associated with energy production, such as mitochondrial and ribosomal genes, increased in expression as the moult cycle progressed. ATP synthase catalyses the synthesis of ATP (the fundamental means of cell energy production) via a proton gradient [84] generated by cytochrome oxidases and NADH dehydrogenase which are the proton translocating enzymes of the mitochondria [85]. Arginine kinase and fumerase are also involved in cell metabolism and energy production, where arginine kinase plays a role in the maintenance of ATP levels in cells with fluctuating energy requirements [37], while fumarase is a catalyst in the Krebs Cycle and has also been associated with growth and development. Here we find an increase in these metabolic transcripts across consecutive stages of the moult cycle with a peak in pre-moult. This may reflect greater physical and/or biological activity in the animals in comparison to their relative sedentary state after moulting occurs, and also greater metabolic demands due to animal growth and new cuticle formation.

A number of genes likely to play an important role in the formation and hardening of the crustacean exoskeleton, such as cuticle proteins, PO activators, lectins, fatty acid-binding proteins and members of the hemocyanin family, have been identified by virtue of protein domain annotation and differential gene expression data. These genes display expression profiles specific to function across the moult cycle. Temporal variation in expression has even been observed between individual cuticular protein transcripts containing the same protein domains (such as chitin or calcium binding). This suggests a difference in functionality for each gene, indicating that transcripts from a similar group may play a distinct and different role in the formation of the crustacean exoskeleton.

Glycosylation of cuticular proteins in the crustacean exoskeleton has been implicated in the regulation of cuticle calcification [73,86]. The recognition of glycosylation sites by mannose-binding lectins is also involved in the activation of serine proteases, which in turn activates the PO cascade [76]. Considering the involvement of POs in the sclerotization of the arthropod cuticle, and the potential role of mannose binding protein in the initiation of mineralisation, the specific up-regulation of mannose-binding protein exclusively during these stages indicates that it plays an important role in exoskeletal hardening. Additionally, transcripts potentially involved in the deposition of lipids in the newly forming cuticle of crustaceans, were up-regulated in the pre-moult stage of *P. pelagicus*.

A large diversity of genes representing many important biological functions related to moulting in crustaceans were able to annotated, and their expression profiles mapped across consecutive stages of the moult cycle of *P. pelagicus *in a time series manner. This approach aims to enhance the knowledge of the molecular mechanisms and regulating factors involved in the moult cycle, and allows the identification of target genes which may control important aspects of various stages of the moult cycle.

## Methods

### Animal selection

*P. pelagicus *crabs were supplied by staff at the Department of Employment, Economic Development and Innovation, Bribie Island Research Centre (BIRC). The crabs were individually housed in a flowthrough system at an ambient water temperature of 24°C, and fed a commercial diet (Ebistar, Higashimaru, Japan) twice daily. Two size groups of crabs were used, small crabs of an average carapace width of 4 cm, and larger crabs of an average carapace width of 11 cm. All crabs were moult staged by examination of pleopod paddles for epidermal retraction and grouped into the following moult stages; moult (shedding of the exoskeleton), post-moult (pliable exoskeleton), intermoult (hard exoskeleton with no evidence of epidermal retraction) early and late stage pre-moult (based on the extent of epidermal retraction) [87].

### cDNA library construction

Two cDNA libraries were constructed using various source tissues, selected in order to provide a diverse collection of transcripts, and representing a broad range of tissue functions and physiological states in all moult stages. One of the cDNA libraries was synthesised from whole animals in order to obtain transcripts from each tissue type. For this library, six small crabs, from each of the following five moult stages; moult, post-moult, intermoult, early and late pre-moult stages, were selected, snap frozen and individually ground under liquid nitrogen. The second cDNA library was derived from organs previously identified as being important to the moult cycle of crustaceans and served to enrich the array with sequences particularly relevant to crustacean moulting. The tissues represented in the *P. pelagicus *organ library were brain, eyestalk, mandibular organ (MO) and Y-organ. These tissues were obtained from six anaesthetised large *P. pelagicus *crabs from each of moult, post-moult, intermoult, and early and late pre-moult stages, and stored in RNA later (Ambion, Austin, USA).

Total RNA was purified from each sample using TRIZOL reagent as recommended by the manufacturer (Invitrogen Life Technologies, Carlsbad, CA, USA). Concentration and purity of the RNA were determined using a spectrophotometer (GeneQuant Pro, GE Healthcare UK Ltd., Buckinghamshire, England) with 260 and 280 nm readings. RNA quality was assessed for all samples by visualisation on a denaturing formaldehyde RNA gel (protocol recommended by Qiagen, Valencia, CA, USA) and ethidium bromide staining. Each cDNA library was constructed by pooling equal amounts of total RNA from all moult cycle stages.

A commercial cDNA library synthesis system (SMART cDNA library construction kit, Clonetech, Mountain View, CA, USA) was used for the construction of each library according to the manufacturer's instructions. Only the final cloning step was modified so that instead of using the λ TriplEx2 vector supplied with the kit, the size fractionated cDNA was ligated into pGEM-T Easy (Promega, Madison, WI, USA) according to the manufacturer's instructions, and transformed into XL10 Gold ultracompetent cells (Stratagene, La Jolla, CA, USA) according to the manufacturer's protocol. 80 clones, randomly selected from each library, were then sequenced and analysed using BLAST http://www.ncbi.nlm.nih.gov/BLAST/ to determine transcript identity and redundancy. The primer used for sequencing was the 5'SMARTlibPCR primer (5'-AAGCAGTGGTATCAACGCAGAGT-3') a modification of the SMART IV oligonucleotide supplied with the SMART cDNA library construction kit (Clonetech).

### Screening for redundant clones

Upon examination of the sequences of 160 clones, from the cDNA libraries of both whole crab and crab organ, redundancies for 16 S ribosomal RNA (rRNA) transcripts were found to be as high as 30%. To remove 16 S rRNA carrying plasmids, all of the clones were first screened for the 16 S rRNA sequence, using a colony hybridisation method [88]. Briefly three probes, (500 bp, 344 bp and 300 bp in length) were designed from separate regions of the 16 S rRNA sequence. These probes were PCR amplified and labelled with ^32^P, then hybridised to clones that had been fixed to nitrocellulose filters. Following an overnight incubation at 55°C in hybridisation buffer (6xSSC and 1% SDS), the filters were washed twice at 55°C in a solution of 6xSSC and 0.2% SDS for 30 min, sealed within plastic and exposed onto autoradiography films (GE Healthcare UK Ltd.) at -70°C using intensifying screens. The films were then developed according to supplier's instructions.

### Construction of custom *P. pelagicus *cDNA microarrays

5000 unsequenced clones, that had been pre-screened for 16 S rRNA, were randomly selected for spotting onto the microarray slides. 2400 were selected from the whole crab library and 2600 from the crab organ library. These were grown overnight in LB containing 50 μg/ml ampicillin. The clones were sent to the AgGenomics (Bundoora, Vic, Australia) microarray printing facility. The clones were PCR amplified using kit-supplied primers (Clontech) and contact-spotted (in duplicate) using pins, onto amino silane coated glass slides, in a 50% DMSO buffer. Known crab genes, that were identified at the initial sequencing stage, such as actin (GenBank accession EF110528) cryptocyanin (EF102021), hemocyanin (EF110534), metallothionein (EF110529), opsin (EF110527) and ubiquitin (EF110526) were spotted onto the arrays for use as controls. Genes specifically associated with the moulting process such as moult-inhibiting hormone (MIH) (EF110524), crustacean hyperglycaemic hormone (CHH) (EF110525) and FaMeT long isoform (DQ085282) [89], were isolated separately from *P. pelagicus *through the design of gene specific primers and spotted on to the arrays. In addition universal reference RNA standard controls (Lucidea, GE Healthcare UK Ltd.) were also spotted onto each array, as were negative control spots of 50% DMSO (without cDNA). The cDNA was bound to the slide surface by baking and UV crosslinking.

### Experimental Design

In order to identify differential gene expression across moult stages, two consecutive moult stages were compared on each array in a dual colour (Cy3 and Cy5) experiment. RNA samples (isolated from individual crabs) were pooled across subjects in order to reduce the effect of biological variation. A formula, that dictates the total number of subjects and arrays required for the pooled experiment to obtain gene expression estimates and confidence intervals comparable to those obtained from a non-pooled experiment [90], gave 90% confidence if nine subjects were pooled across a total of three arrays. To this effect, equal amounts of total RNA from three crabs in one moult stage, were pooled, and compared against equal amounts of total RNA pooled from three crabs in another moult stage, on one array. This was repeated three times in total, the different moult stages were labelled with Cy3 or Cy5 respectively. Consecutive moult stages were compared in the following format; post-moult (Cy3) with intermoult (Cy5), intermoult (Cy3) with early pre-moult (Cy5), early pre-moult (Cy3) with late pre-moult (Cy5), late pre-moult (Cy3) with ecdysis (Cy5), and ecdysis (Cy3) with post-moult (Cy5). Figure 2 is a schematic diagram depicting each set of moult stage comparisons.

Spatial variation within each array was addressed through spot duplication. Two identical blocks of grids consisting of each amplified cDNA and including the controls described above were printed onto the left and right sides of each horizontally orientated array, thus affording spatial separation between duplicate spots, to allow for the normalisation of potential hybridisation anomalies.

### Microarray Hybridisations

Nine small crabs (six of these were also used in the above described whole crab cDNA library construction) were snap frozen, individually ground under liquid nitrogen and RNA was isolated from each ground crab using TRIZOL reagent as recommended by the manufacturer (Invitrogen Life Technologies). The RNA was DNase treated using RQ1 RNase free DNase (Promega) according to the manufacturer's instructions and purified using RNeasy Mini Kit (Qiagen) as recommended by the manufacturer. RNA quality was assessed by visualisation on a denaturing formaldehyde RNA gel (protocol recommended by Qiagen) using ethidium bromide staining. Concentration and purity of the RNA were determined by measuring the absorbance at 260 nm and 280 nm using a spectrophotometer (GeneQuant Pro). One microgram of Lucidea universal RNA control (GE Healthcare) was added to 10 μg of pooled total RNA for each moult stage sample, the RNA was converted to cDNA then labelled and hybridised to the array using the 3DNA Array 900 MPX expression array detection kit (Genisphere Inc., Hatfield, PA, USA) according to the manufacturer's protocol. Briefly, RNA was reverse transcribed using a random primer combined with an oligo dT primer. The RNA was then degraded and the cDNA tailed with dTTP followed by ligation to a dendrimer-specific capture oligo (specific for either Cy3 or Cy5). Microarray slides were denatured prior to use by immersion in 95°C MilliQ water for 5 min, the slides were then transferred to 95% ethanol at room temperature for 2 min. Slides were spun dry to reduce streaking at 800 RPM for 2 min. The Cy3 and Cy5 "tagged" cDNAs were combined and then hybridised to the array by overnight incubation in a humidity chamber at 65°C using the kit supplied non-formamide SDS-based buffer and a poly T based blocker, according to the manufacturer's specifications. The "tagged" cDNA was washed with a series of three SSC-based buffers, the first wash occurred at 65°C for 15 min, the other wash steps were carried out at room temperature for 10 min each. The slides were spun dry at 800 RPM for 2 minutes. Fluorescent 3DNA capture reagent (which carries a sequence complementary to the Cy3 and Cy5 tag) was then hybridised to the array using the SDS-based buffer with added Anti-Fade reagent (inhibits photobleaching of Cy5) at 65°C for four hrs. The fluorescent reagent was then washed as described above for the cDNA hybridisation.

### Data analysis

Microarray slides were scanned using a white-light ArrayWorx Biochip Reader (Applied Precision, LLC, Issaquah, Washington, USA). ImaGene (BioDiscovery Inc., El Segundo, CA, USA) was then used to process images and create spot intensity reports, while CloneTracker (Biodiscovery Inc.) was used to generate gene ID mapping files and assign gene identification. Final intensity reports were retrieved as raw spot intensities in tab-delimited files. The data set is deposited in the Gene Expression Omnibus (GEO) database (accession no. GSE6997) at the following site: http://www.ncbi.nlm.nih.gov/geo/.

Microarray data analysis was performed using GeneSpring GX 11.0 (Agilent Technologies Inc., Santa Clara, CA, USA). The single colour workflow feature of GeneSpring GX was used in order to split the two channel array into 2 single colour experiments to enable the analysis of multiple samples across different arrays. Using the loop design depicted in Figure 2 a comparison across the moult cycle was made by creating a 'time series plot' with each point representing a particular moult stage.

The two colour data was normalised using the robust scatter plot smoother LOESS (also known as "LOWESS" for locally-weighted regression and smoothing scatter plots) [91]. For each chip, normalisation was applied to the left and right sides separately (spatial positioning of clones spotted in duplicate was in the format of two grids located on the left and right side of each array when orientated horizontally). Raw signals were thresholded to 1.0 and an additional normalisation using the percentile shift algorithm to the 75^th ^percentile was used. Since each feature is spotted onto an array in duplicate, and three biological replicates are performed per moult stage comparison, a standard error, a *t*-statistic, and *t*-distribution (*P *value) can be calculated for each feature represented on the array. K-Means clustering was employed to group transcripts with similar expression profiles together. The Euclidean distance measure was used, which takes the standard sum of squared distance between two entities.

### Sequence and phylogenetic analysis

Following hybridisation experiments, clones that displayed differential expression (P ≤ 0.05) patterns across moult stages were sequenced. Overlapping sequences (contigs), that likely represent the same cDNA, and clones without sequence identity to other cDNAs (singletons) were identified by comparing all sequences against one another in Sequencher (Gene Codes Corporation, Ann Arbor, MI, USA). The genes were annotated with the name of the highest basic local alignment search tool (BLAST) [92] score from an analysis of GenBank entries by the BLASTx and BLASTn procedures. Protein domains were identified from the Pfam database [93], and InterProScan http://www.ebi.ac.uk/InterProScan.

## Authors' contributions

AK performed the moult staging, animal husbandry and sampling, library preparation, sequencing, bioinformatic analysis of the ESTs, microarray construction, microarray hybridizations, and interpretation of the data, contributed to the experimental design and drafted the manuscript. TH contributed to the construction of the microarrays, the microarray hybridizations and analysis. BP contributed to the conception and design of the study and provided the experimental animals. AE contributed to the conception and design of the study, coordinated the work, data interpretation and drafting of the manuscript. All authors read and approved the final manuscript.
